# High Magnesium Corrosion Rate has an Effect on Osteoclast and Mesenchymal Stem Cell Role During Bone Remodelling

**DOI:** 10.1038/s41598-018-28476-w

**Published:** 2018-07-03

**Authors:** Diana Maradze, David Musson, Yufeng Zheng, Jillian Cornish, Mark Lewis, Yang Liu

**Affiliations:** 1 Centre of Biological Engineering, Wolfson School of Mechanical, Electrical and Manufacturing Engineering, Loughborough University, Loughborough, Leicestershire LE11 3TU UK; 2 Department of Medicine, University of Auckland, Auckland, New Zealand; 3 College of Engineering, Peking University, Beijing, 100871 China; 4 School of Sport, Exercise and Health Sciences, Loughborough University, Loughborough, Leicestershire LE11 3TU UK

## Abstract

The aim of this study was to gain an understanding on the collective cellular effects of magnesium (Mg) corrosion products on the behaviour of cells responsible for bone formation and remodelling. The response of mesenchymal stem cells (MSCs) and osteoclast cells to both soluble (Mg ions) and insoluble (granule) corrosion products were recapitulated *in vitro* by controlling the concentration of the corrosion products. Clearance of corrosion granules by MSCs was also inspected by TEM analysis at sub-cellular level. The effect of Mg corrosion products varied depending on the state of differentiation of cells, concentration and length of exposure. The presence of the corrosion products significantly altered the cells’ metabolic and proliferative activities, which further affected cell fusion/differentiation. While cells tolerated higher than physiological range of Mg concentration (16 mM), concentrations below 10 mM were beneficial for cell growth. Furthermore, MSCs were shown to contribute to the clearance of intercellular corrosion granules, whilst high concentrations of corrosion products negatively impacted on osteoclast progenitor cell number and mature osteoclast cell function.

## Introduction

**Figure 1:**
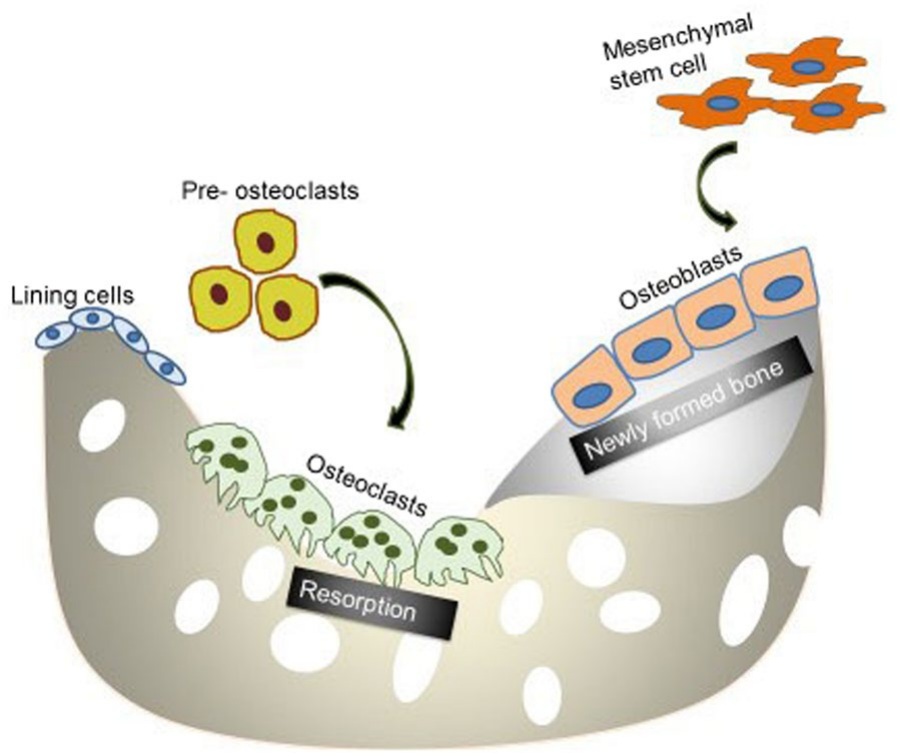
Bone Remodelling Process. Activation of remodelling is initiated when bone lining cells separate to expose bone and pre-osteoclast cells are recruited to the site. Mature osteoclast resorb the old bone and mature osteoblast lay down new bone.

Current orthopaedic implants include the use of metallic biomaterials, ceramics and polymers. Currently approved metallic biomaterials include stainless steel, cobalt-chromium alloys and titanium based alloys. Limitations of using these inert materials include possible release of toxic wear particles to the surrounding tissues. The elastic moduli of these metals are not matched with that of bone, leading to stress shielding effects and ultimately result in reduction of bone formation and remodelling^1^. Biodegradable Mg has an elastic modulus closer to that of bone, and as such, its use as biomaterial for orthopaedic implant reduces the likelihood of stress shielding. As Mg corrodes *in vivo* it aids biological repair and simultaneously becomes less important as a constituent for mechanical support. Mg also plays an important role in a number of biological functions and is involved in bone and mineral homeostasis. Bone is remodelled to maintain strength and mineral homeostasis. During remodelling, osteoclasts remove old bone and osteoblasts lay down new bone to prevent accumulation of micro-damage (Fig. 1)^2,3^.

As Mg degrades at the implantation site there is subsequent release of large particulate material and smaller corrosion products. Relatively few studies have detailed effects of Mg corrosion on progenitor cells at the implantation site. The ability of the body to clear the granules from the implantation site is crucial for tissue implant integration. While some studies^4–6^ have reported enhanced bone formation near the implantation site, others^7,8^ have demonstrated the presence of cavities in the implant position after the Mg implant had degraded. The cause of these cavities remains uncertain. It has been suggested the presence of the granules might attract the migration of osteoclasts to the implantation site^9^; and subsequent increased activity of the osteoclast could aid bone remodelling. Incidentally, overactive osteoclast activity could also lead to an unbalanced remodelling processes resulting in the formation of bone cavities at the implantation site.

It is therefore imperative to have a fundamental understanding of Mg corrosion products effect on not only osteoblast but also osteoclast activity and function. Alterations in the functions of these cells could offset bone homeostasis leading to the development of bone disease or impairment of bone healing. It is against this backdrop that the study was undertaken to get a better understanding of the collective cellular effects of Mg corrosion products on the behaviour of various cell types responsible for bone formation and remodelling. The spatial and temporal factors of tissue response were recapitulated *in vitro* by controlling the concentration of the corrosion products.

## Materials and Methods

### Mg Sample Preparation

Commercial pure Mg (99.9%) in the form of cylindrical ingots was supplied by a partner from Peking University, Beijing, China. The Mg disks were sterilised by soaking them in 100% (v/v) ethanol for 5 mins and were subsequently irradiated under ultraviolet light (UV) for 3 hours each side. Mg disks had average measurements of 12.2 mm diameter and 4.75 mm depth and weighed approximately 1 g each.

### Preparation of Mg corrosion products *in vitro*

**Figure 2:**
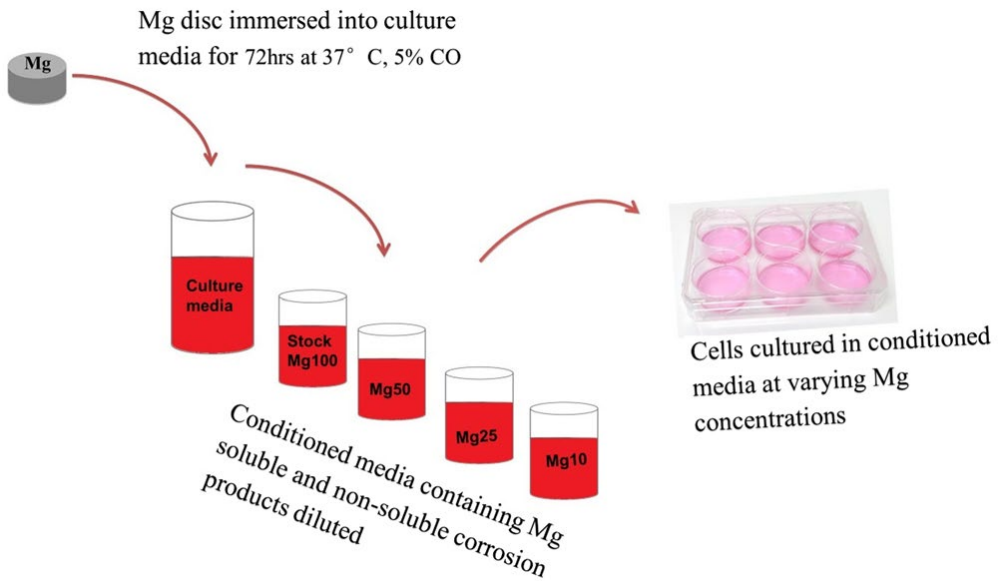
Preparation of Mg conditioned media *in vitro*.

Mg disk was immersed in 400 ml of appropriate cell culture medium for the specific cell type and kept in a humidified atmosphere with 5% CO_2_ for 72 hours. In order to distinguish between the effects of released corrosion products presented as insoluble granules and soluble ions, part of the conditioned medium was filtered using a 0.22 μm sterile filter (Millipore, UK) to prepare a condition medium free of particulate (filtered medium), while the other half was left unfiltered (non-filtered medium). The stock conditioned medium (100) was diluted 1:2 (50), 1:4 (25) and 1:10 (10) with fresh culture medium and then supplemented with growth supplements as required for culture of the specific cell type (Fig. 2). Inductively coupled plasma optical emission spectrometry (ICP-OES), (iCAP 6000 series, ThermoFisher Scientific) was used to quantify the amount of Mg^2+^ in the conditioned media.

### Chemical Analysis of Corrosion Granules

A separate analysis was performed to measure the amount of corrosion granules present following corrosion in culture medium. After a 72 hour immersion in the appropriate culture medium, the resultant conditioned medium was centrifuged at 800 g. The supernatant was removed and the corrosion granules were allowed to dry before the weight was measured. The amount of the corrosion granules formed was measured at approximately 1.5 mg/ml for the non-filtered undiluted concentration (Mg100) and the pH was measured at 8.5 using a pH meter. Scanning electron microscopy (SEM) and energy dispersive X-ray (EDX) was then conducted for chemical analysis of the corrosion granules.

### Cell Culture

All the cells were cultured *in vitro* at 37 °C, 5% CO_2_. MSC growth medium comprised of Dulbecco’s Modified Eagle’s Medium (DMEM) (Lonza, UK) supplemented with 10% (v/v) foetal bovine serum (FBS) (Sigma-Aldrich, UK), L-glutamine final media concentration 2 mM (ThermoFisher Scientific, UK), and 100 units/ml penicillin-streptomycin (ThermoFisher Scientific, UK). MSC osteogenic medium comprised of MSC growth media supplemented with 100 nM dexamethasone (Sigma Aldrich, UK), 10 mM glycerolphosphate (Sigma Aldrich, UK) and 50 μg/ml L-ascorbic acid (Sigma Aldrich, UK). RAW growth medium comprised of α-MEM (Life Technologies, NZ) supplemented with 10% (v/v) FBS (Life Technologies, NZ), L-glutamine final media concentration 2 mM (Life Technologies, NZ) and 100 units/ml penicillin-streptomycin (Life Technologies, NZ). RAW cell differentiation medium comprised of growth media supplemented with 10 ng/ml RANK-L (Amgen). Mature osteoclast (MO) growth medium comprised of Earle’s MEM (ThermoFisher Scientific, NZ) supplemented with 10% (v/v) FBS, 100 units/ml penicillin-streptomycin and 0.1% 12 M HCL.

### Measurement of Cell Viability

Human bone marrow derived MSCs (hMSCs) (Lonza, USA) were seeded onto a 24 well plate at a density of 10 000 cells/well in triplicate. Cells were incubated in MSC growth medium for 24 hours to allow for attachment. The MSC growth medium was then replaced with Mg filtered medium or non-filtered medium (DMEM) supplemented with 10% (v/v) FBS, L-glutamine final media concentration 2 mM, and 100 units/ml penicillin-streptomycin. Cells cultured in MSC growth medium were used as the standard control. Viability assay was performed after cells were cultured with conditioned medium on day 1, 3 and 7 using AlamarBlue reagent (Invitrogen, Life Technologies). The same procedures were also carried out for RAW cells (ATCC *In vitro* technologies, NZ) and the viability was measured on day 1, 2 and 3.

AlamarBlue assay was performed following manufacturer’s protocol. Fluorescence level of the supernatant was measured at excitation: emission 530:590 nm and normalised to DNA concentration (PicoGreen assay). Fluorescence level of growth medium without cells was used as the background control. Fluorescence level from the background was subtracted from the fluorescence level of the treated and standard control sample. Cell viability ≥70% relative to the control was defined as a non-toxic effect; this is in accordance to the ISO standard for biological evaluation of medical devices (ISO 10993-5:2009).

### DNA Quantification

DNA was quantified using the Quant-iT PicoGreen Kit (Invitrogen USA) following manufacturers’ protocol. Briefly, cells were lysed with trypsin and 0.1% Triton X at a ratio of 1:1. 100 μL of each cell lysate solution was added to a black 96 well plate and 100 μL of Quanti-iT PicoGreen reagent (Life Technologies) was added to each well and incubated in the dark for 5 minutes. Fluorescent intensity was read using the plate reader at excitation: emission wavelength of 480 nm:520 nm.

### Osteogenic Differentiation of hMSCs

hMSCs were seeded onto a 6 well plate at a density of 60 000 cells/well in triplicate. Cells were incubated in MSC growth medium until 90% confluent. The MSC growth medium was then replaced with Mg filtered medium or non-filtered medium (DMEM) supplemented with 10% (v/v) FBS, L-glutamine final media concentration 2 mM, and 100 units/ml penicillin-streptomycin and cells were cultured for a period of 12 days. Cells cultured in MSC growth medium were used as the standard control and cells cultured in osteogenic medium were used as the positive control. RNA was extracted at day 2, 4, 8 and 12 using RNeasy Kit (QIAGEN, UK) as per manufacturers’ instructions. The quantity and purity of RNA was measured using a Nanodrop spectrophotometer (Themo Fisher Scientific). The RNA produced was used for quantitative real time PCR (qRT-PCR) to amplify the primers of osteogenesis-related genes: Runx-2, collagen type 1-alpha 1 (COL1A1) and osteocalcin (Quantitect primer assays, QIAGEN, UK) using a one-step real time PCR machine (ViiA 7, Applied Biosystems) and QuantiFast SYBR Green RT-PCR Kit (QIAGEN, UK). GAPDH was used as the endogenous control. Amplification was performed for 40 cycles consisting of reverse transcription at 50 °C for 10 minutes, DNA polymerase activation at 95 °C for 5 minutes, denaturation at 95 °C for 10 seconds and annealing/extension at 60 °C for 30 seconds. Melting curves were generated to distinguish and exclude non-specific amplifications and primer dimers. Ct values were analysed by the comparative Ct (ΔΔCt) method. Ct value of each sample was normalised with the average Ct value of the endogenous control (GAPDH). The fold change in gene expression was calculated as 2(^−ΔΔCt^).

### Alkaline Phosphatase (ALP) Activity

hMSCs were seeded onto a 24 well plate at a density of 10 000 cells/well in triplicate. Cells were incubated in MSC growth medium until 90% confluent. The MSC growth medium was then replaced with Mg filtered medium or non-filtered medium (DMEM) supplemented with 10% (v/v) FBS, L-glutamine final media concentration 2 mM, and 100 units/ml penicillin-streptomycin and cultured for a period of 12 days. Cells cultured in MSC growth medium were used as the standard control and cells cultured in osteogenic medium were used as the positive control. ALP assay was performed at day 2, 7 and 12 using p-Nitrophenyl Phosphate (pNPP) Liquid Substrate System, (Sigma Aldrich, UK). Briefly, cells were lysed in 0.1% Triton X-100; pNPP solution was added to each well and the plate was incubated in the dark for approximately 30 minutes at room temperature. The production of p-nitrophenol (PNP) was quantified at an absorbance of 405 nm using a plate reader and normalised to DNA concentration.

### Osteoclastogenesis of RAW cells

Mouse RAW (264.7) cells from a transformed macrophage cell line (ATCC, The Global Bioresource Centre, NZ) were used for experimental procedures. In the presence of RANK-L these cells can be induced to differentiate into osteoclast-like cells. RAW cells were seeded onto a 48 well plate at a density of 4000 cells/well, 8 wells were used for each condition. Cells were incubated in RAW growth medium for 3 hours. After 3 hours, RAW growth medium was then replaced with Mg filtered medium or non-filtered medium (α-MEM) supplemented with 10% (v/v) FBS, L-glutamine final media concentration 2 mM, 100 units/ml penicillin-streptomycin and 10 ng/mL RANK-L. The cells were allowed to differentiate over a culture period of 5 days. RNA was extracted and quantified as mentioned previously. Cells cultured in RAW growth medium were used as the standard control and cells cultured in RAW differentiation medium were used as the positive control. In comparison to the hMSCs, a two-step PCR process was used to produce and amplify cDNA of osteoclastogenesis related genes using a real time PCR machine (QuantStudio 12 K, Applied Biosystems). FAM-labelled TaqMan assays specific for NFATc-1 and TRAP and VIC-labelled 18 S rRNA endogenous control were used according to manufacturers’ instructions (ThermoFisher Scientific). SuperScript III first-strand synthesis system (Invitrogen, Life Technologies) was used for the reverse transcription of RNA into cDNA according to manufacturer’s protocol. Amplification of the cDNA was performed for 40 cycles, DNA polymerase activation at 95 °C for 10 mins, denaturation at 95 °C for 15 seconds and annealing/extension at 60 °C for 1 min. Ct values were analysed by the comparative Ct (ΔΔCt) method. Ct value of each sample was normalised with the average Ct value of the endogenous control (18 S rRNA). The fold change in gene expression was calculated as 2(^−ΔΔCt^).

### TRAP Staining

RAW cells were seeded onto a 48 well plate at a density of 2000 cells/well, 8 wells were used for each sample. Cells were incubated in RAW growth medium for 3 hrs. After 3 hrs, RAW growth medium was then replaced with Mg filtered medium or non-filtered medium (α-MEM) supplemented with 10% (v/v) FBS, L-glutamine final media concentration 2 mM, 100 units/ml penicillin-streptomycin and 10 ng/mL RANK-L. The cells were allowed to differentiate over a culture period of 5 days. Cells were fixed using 200 μL of TRAP fixative (25% Sodium citrate (0.01 M) +65% Acetone +40% formaldehyde) for 30 secs. Cells were stained for TRAP using a Leukocyte Acid Phosphatase kit (Sigma Aldrich) following manufactures’ instructions.

### Mature Osteoclast: substrate preparation and cell isolation

32 dentin bone slices (supplied by the University of Auckland) were used as cell culture substrates for mature osteoclasts. The bone slices were sonicated in distilled water for 5-10 mins and then sterilized with 70% ethanol for 10 mins followed by a rinse in PBS and then RAW growth medium. The slices were placed into 96-well plate and 50 μl RAW growth medium was added to each of the wells. To prepare Mg conditioned medium containing various concentrations of corrosion products, Mg non-filtered medium (RPMI 1640) supplemented with 10% (v/v) FBS, L-glutamine final media concentration 2 mM and 100 units/ml penicillin-streptomycin was added to a 12 well plate. The 96-well plate with the bone slices and the 12-well plate with Mg non-filtered medium were placed in a humidified atmosphere at 37 °C under 5% CO_2_. Mature osteoclast cells were isolated as previously described by Cornish *et al*.^10^. The osteoclast cell suspension was then loaded (150–180 μl/well) onto the 96-well plate containing the bone slices and incubated for 20–30 mins in a humidified atmosphere at 37 °C under 5% CO_2_. After 30 mins the bone slices were rinsed twice with PBS to remove any contaminating non-osteoclastic cells and then transferred to the previously prepared 12 well plate containing Mg conditioned medium. Cells cultured in MO growth medium were used as the standard control. Cells cultured with the presence of 9 M salmon calcitonin (sCT-9M) (Bachem, Inc. Torrance, CA), an inhibitor of osteoclast resorption activity were used as the positive control. After 24 hrs incubation, the bone slices were fixed and mature osteoclast cell number and activity was determined as previously described by Cornish *et al*.^10^.

### Live imaging

Live imaging of hMSCs cultured in Mg non-filtered medium was performed using the Cell 1Q imagen, a live cell observation and analysis system equipped with a Nikon microscope (TAP Biosystems). Cells were seeded in a 6 well plate at a density of 60 000 cells/well. The cells were cultured in MSC growth medium for 24 hrs to allow for attachment. After 24 hrs MSC growth medium was replaced with Mg non-filtered medium (DMEM) supplemented with 10% (v/v) FBS, L-glutamine final media concentration 2 mM, and 100 units/ml penicillin-streptomycin. The cells were incubated in the Cell IQ system at 37 °C and 5% CO_2_ under humidified conditions. Images were taken at 7 different locations of each well every 10 minutes for 48 hrs; video files were also constructed from the images.

### Transmission electron microscopy (TEM)

hMSCs were seeded in a 6 well plate at a density of 60 000 cells/well. The cells were cultured in MSC growth medium for 24 hrs to allow for attachment. After 24 hrs, MSC growth medium was replaced with Mg non-filtered medium (DMEM) supplemented with 10% (v/v) FBS, L-glutamine final media concentration 2 mM, and 100 units/ml penicillin-streptomycin. After 48 hrs, cells were harvested using trypsin and fixed in 3% glutaraldehyde for 2 hrs (TAAB Laboratories, UK). Following fixation, the cell suspension was centrifuged at 500 g for 5 mins, fixative was removed and pellet was resuspended in 0.1 M cacodylate buffer (TAAB Laboratories, UK). The cell pellet was then fixed in 1% aqueous osmium tetroxide for 30 mins (TAAB Laboratories, UK), followed by dehydration in a series of graded concentration of ethanol, 10 mins each, followed by 100% Propylene Oxide (propox) (TAAB laboratories, UK) for 15 mins. Araldite CY212 resin (TAAB Laboratories, UK) was prepared according to manufactures’ instructions. Following dehydration the cell pellet was infiltrated with resin (1:3 resin: propox mix for 2 hrs, 1:1 resin: propox mix overnight and then in pure resin for 2 hrs). The pellet was then embedded in a BEEM capsule and left in the embedding oven at 60 °C for at least 48 hrs. After 48 hrs the embedded cell pellet was cut into 90 nm thick samples using an ultramicrotome. The samples were then placed on TEM support mesh grids (Agar Scientific) and stained with uranyl acetate followed by Reynold’s lead citrate (TAAB laboratories, UK) for 5 mins. TEM images were taken using the Tecnai BioTwin-12 TEM (FEI, USA).

### Statistical Analysis

Average and standard deviations were calculated from repeats on each sample, where n represents the number of repeats for each sample. Error bars on graphs represent the mean and standard deviation in the positive orientation of three independent experiments. Statistical analysis was performed using SPSS software (IBM, USA). Multivariate analysis of variance and one-way ANOVA, including Tukey’s post hoc tests, were performed to detect significant effects of the experimental variables. Significance levels were defined as *p < 0.05, **p < 0.01 and #p < 0.001.

## Results

### Chemical analysis of corrosion products in Mg conditioned media

ICP-OES was used to measure the amount of Mg^2+^ present in the Mg conditioned medium following the corrosion of Mg *in vitro*. The stock/undiluted conditioned medium (Mg100) was used to prepare a series of dilution of 1:2 (Mg50), 1:4 (Mg25) and 1:10 (Mg10) with fresh culture medium. As indicated in Table 1, the dilution of the conditioned media resulted in the concentration of Mg^2+^ being reduced.

**Table 1:** Concentration of Mg ions in the conditioned media following the corrosion of pure magnesium.

Ion concentration (mean ± SD)	
Sample	Mg (mM)
Mg100	15.9 ± 2.8
Mg50	9.9 ± 2.2
Mg25	4.5 ± 0.9
Mg10	2.8 ± 0.9
Control	1.0 ± 0.3

### SEM and EDX analysis of Mg corrosion granules

**Figure 3:**
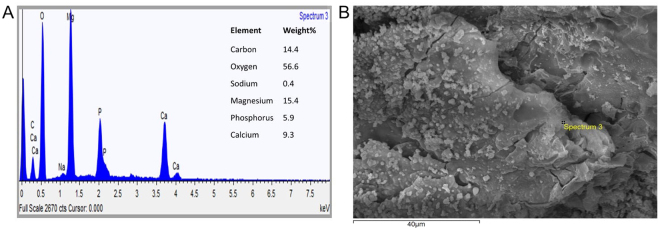
Chemical analysis of Mg corrosion granules after 3 days of corrosion in growth medium under culture conditions. (**A**) SEM analysis of Mg corrosion granules and (**B**) corresponding EDX spectra of Mg corrosion granules.

To gain a better understanding of the corrosion process, methods of chemical and elemental analysis were applied to the solid phase of the corrosion products. Figure 3A illustrates the EDX analysis of Mg corrosion granules after 72 hrs corrosion in culture medium and the corresponding location of analysis is labelled in the SEM image Fig. 3B. EDX analysis revealed the elemental composition of the sample which was found to be C-14%, O-56%, Mg-15%, P-6%, Ca-9%. A ratio of 1.57 Ca/P was derived from the EDX data (Fig. 3). A Ca/P ratio between 1.5 and 1.67 is indicative of calcium deficient hydroxyapatite^11,12^.

### The response of hMSCs to media conditioned with Mg alloy

**Figure 4:**
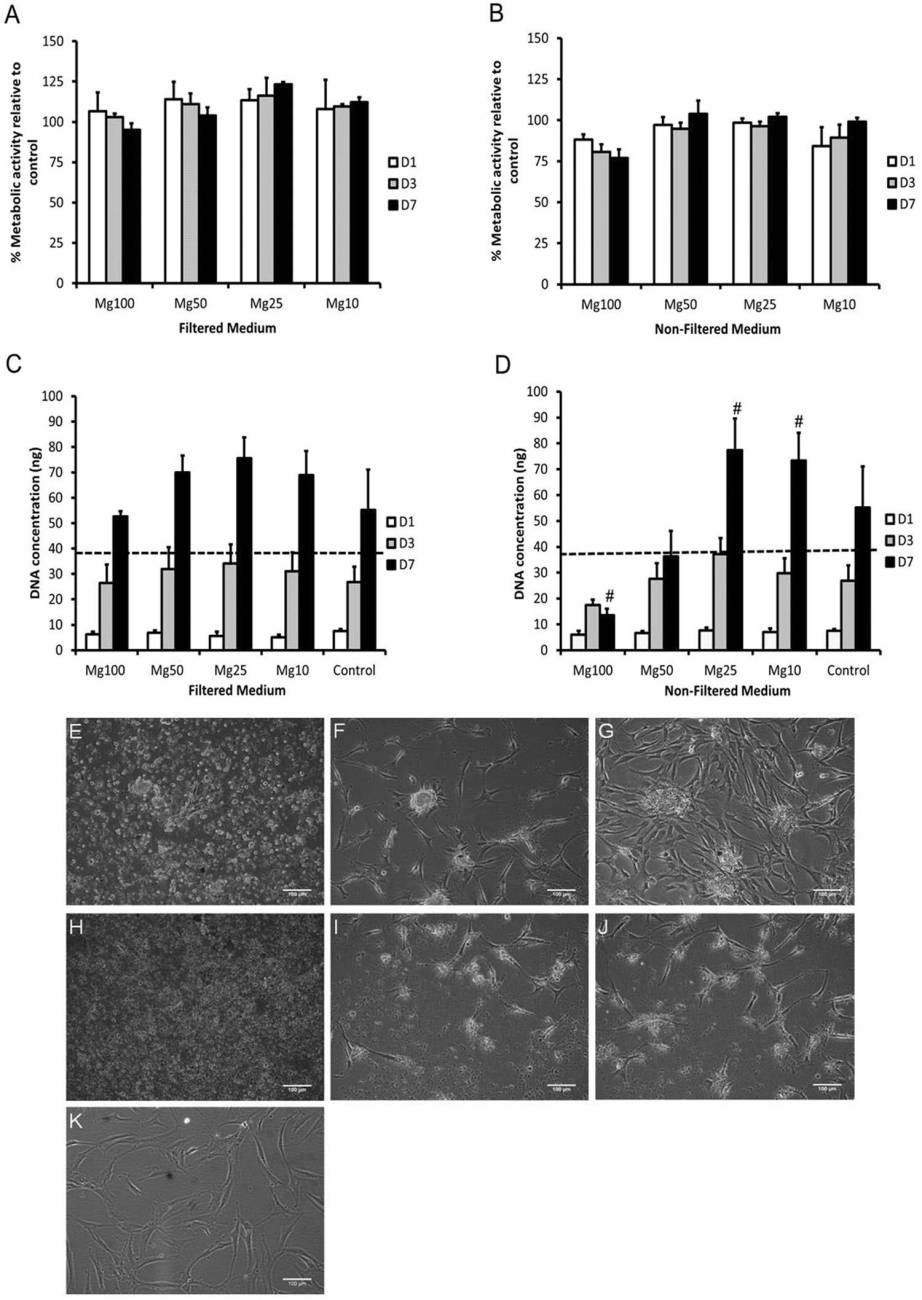
Effect of Mg conditioned media on hMSCs metabolic activity and DNA concentration was investigated using AlamarBlue assay and PicoGreen assay over a period of 7 days. hMSCs were cultured in the presence of various concentrations of Mg conditioned medium, (**A**) filtered medium and (**B**) non-filtered medium. A reduction in metabolic activity was observed when cells were cultured in Mg100 non-filtered medium on day 3 and 7 in comparison to the control. DNA concentration of hMSCs was also investigated following culture in (**C**) filtered medium and (**D**) non-filtered medium. A significant decrease (^#^p < 0.001) in DNA concentration was observed when cells were cultured in Mg100 non-filtered medium on day 7. However, when the Mg non-filtered medium concentration was reduced to Mg25 and Mg10 a significant increase (^#^p < 0.001) in DNA concentration was observed. The data is represented relative to the control (100%). The dotted line represents the cytotoxicity threshold on day 7. The bars represent the mean and standard deviation in the positive orientation of three independent experiments, each with n = 3. Representative images taken using Cell IQ live imaging system shows cells cultured in non-filtered medium (Mg50) at (**E**) 0 hrs (**F**) 24 hrs and (**G**) 48 hrs and (Mg100) at (**H**) 0 hrs (**I**) 24 hrs and (**J**) 48 hrs. (**K**) Image showing cells cultured in standard growth medium (control) at 48 hrs. Scale bar = 100 µm, arrows indicate the presence of cell aggregates.

Figure 4 shows the metabolic activity of cells following culture in the presence of various concentrations of Mg conditioned media. Metabolic activity was above 70% in comparison to the control for all the concentrations (filtered and non-filtered medium) at each time point. Thus it was indicated that hMSCs were able to tolerate Mg concentrations of 15.9 mM. Even in the presence of corrosion granules (non-filtered medium) metabolic activity was ≥70%; however, a decrease in metabolic activity on day 3 and 7 was observed when cells were cultured in Mg100 non-filtered medium compared to the control. On the other hand, cells cultured in Mg25 filtered medium showed an increase in metabolic activity compared to the control on day 7. To quantify the change in cell number, DNA concentration analysis was also performed (Fig. 4C,D). When cells were cultured in filtered medium they were able to proliferate over time (day 1–7), and no cytotoxicity effects were observed. On the other hand, the presence of corrosion granules significantly affected DNA concentration. DNA concentration of cells cultured in Mg100 non-filtered medium significantly (p < 0.001) dropped from day 1 in relation to the control to below the cytotoxicity threshold by day 7 (Fig. 4D); indicating that longer exposure to high concentrations of corrosion granules adversely affects cell proliferation. Indeed, the dilution of Mg100 non-filtered medium to Mg25 and Mg10 reduced the cytotoxicity effects, resulting in a significant (p < 0.001) increase in DNA concentration by day 7. For a different perspective of possible effects of non-filtered medium on cell behaviour, live imaging was performed (see the video in supplemented data). The representative images at 0, 24 and 48 hrs of contact with medium containing corrosion granules are presented in Fig. 4E–K. In the presence of Mg corrosion granules (Mg50 non-filtered medium), cell migration to the granules with subsequent aggregate formation was observed (Fig. 4E–G). No such phenomena were observed when cells were cultured in Mg100 non-filtered medium (Fig. 4H–J) (see video in the supplemented data). Cells cultured in the presence of Mg100 and Mg50 non-filtered appeared to have typical MSC morphology in comparison to the control: the cells had a small cell body with thin and long cell processes.

### TEM analysis of cells treated with Mg conditioned medium

**Figure 5:**
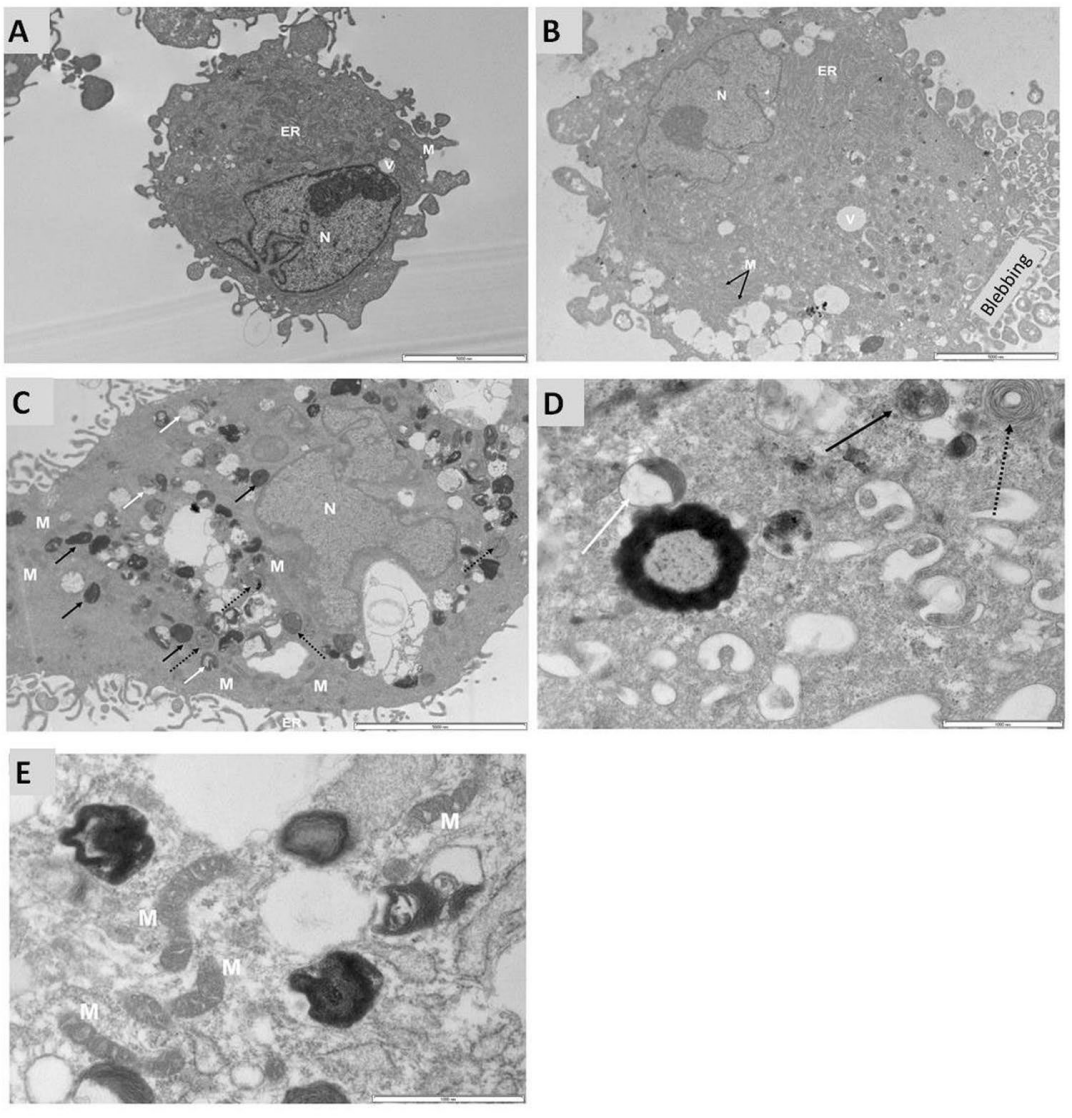
TEM analysis of hMSCs treated with Mg non-filtered medium. (**A**) hMSCs were cultured in standard growth media (control) for 48 hours. The cells showed the presence of normal cellular components, this included a defined nucleus, vacuoles, mitochondria, cisternae of the endoplasmic reticulum scattered within the cytoplasm and an intact membrane. (**B**) By comparison with the control, there was significant blebbing after treatment with Mg non-filtered medium with a few vacuoles and autolysosomes scattered towards the periphery of the cells. ER with moderately dilated cisternae was also observed. (**C**) Formation of different types of autolysosomes was observed following treatment with Mg non-filtered medium. There was an increase in the presence of mitochondria and vacuoles when cells were treated with Mg non-filtered medium. A co-localisation of vacuoles with autolysosomes was also observed following treatment. The arrows are showing the presence of a few autolysosomes within the cell. The white arrow is showing autolysosomes with partially degraded material, and black arrow is showing autolysosomes filled with electron dense material. There was also the presence of autolysosomes filled with concentric lamellar features (broken black arrow). (**D**) Lower magnification image showing the different types of autolysosomes. (**E**) Lower magnification image showing mitochondria scattered among the autolysosomes. Scale bar (Fig. 5A–C) = 5 μm, scale bar (Fig. 5D,E) = 1 μm, M-mitochondria, V- vacuole, N-nucleus, ER-endoplasmic reticulum.

TEM analysis was performed to evaluate the effect of corrosion granules on hMSCs and to verify its presence at subcellular level. hMSCs were cultured in the presence of Mg50 non-filtered medium for 48 hrs; following culture cells were fixed and prepared for TEM analysis. Figure 5A shows cells cultured in standard growth medium (the control). These cells appeared to have normal morphology with intact cell membrane, mitochondria and rough endoplasmic reticulum (ER) structures not altered. 48 hrs after treatment with Mg50 non-filtered medium, the nucleus was still intact with an irregular shape and deep invaginations. The cells became more vacuolated with electron dense filled vacuoles scattered within the cytoplasm (Fig. 5B,C). Significant blebbing was also noticed after treatment with Mg50 non-filtered medium (Fig. 5B). More mitochondria were visible and appeared to be located near the vacuoles (Fig. 5C,F). Different type autolysosomes were observed; the presence of these lysosomes was higher compared to the control (Fig. 5B,C). Features filled with concentric lamellar were observed after cells had been treated with Mg non-filtered medium (Fig. 5C,D). These features appeared to be scattered towards the periphery of the cell. Mitochondria and dilated ER cisternae were also observed around or near these features (Fig. 5C,E).

### The effect of Mg conditioned media on hMSCs gene and protein level

**Figure 6:**
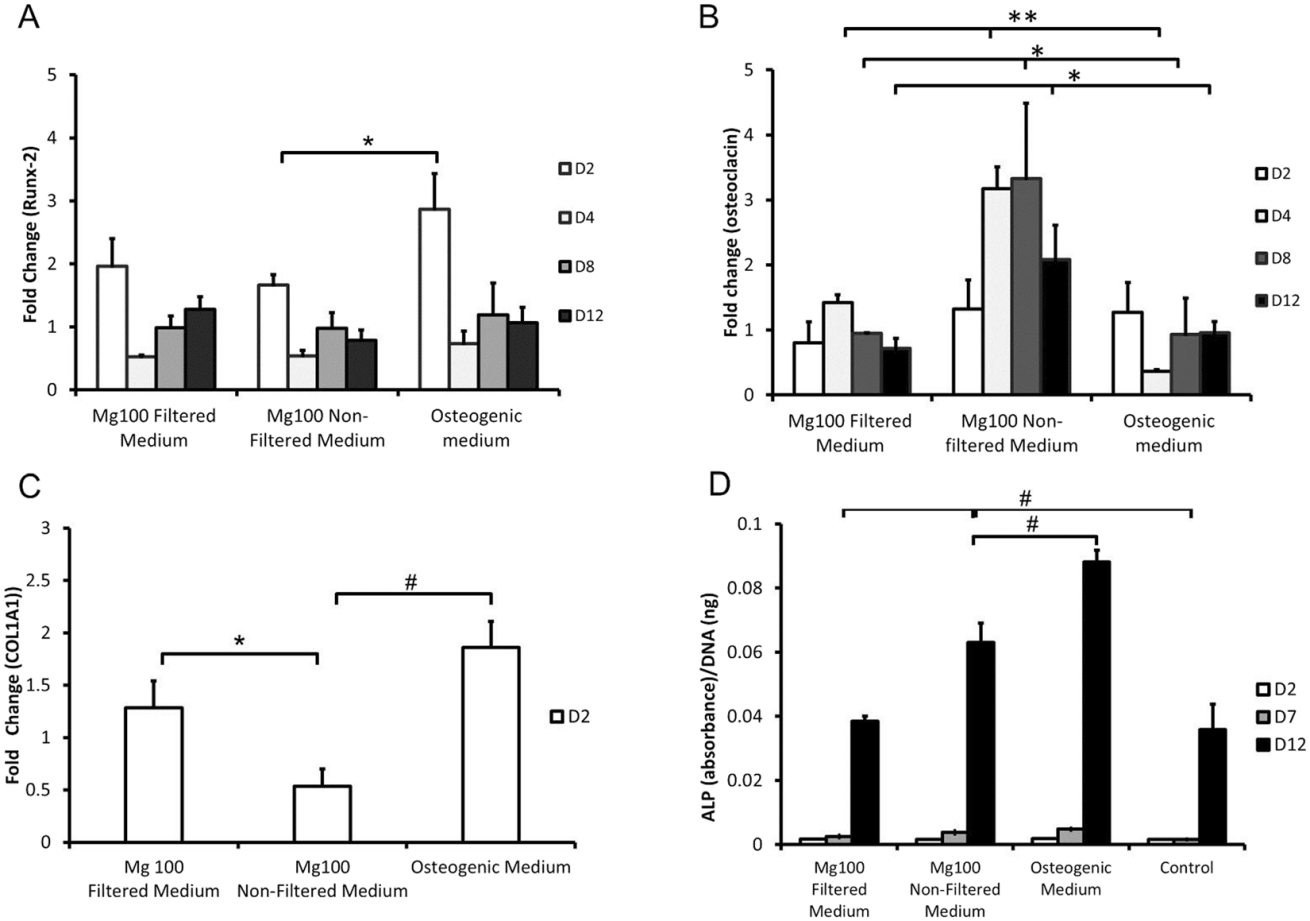
The effect of Mg100 conditioned media on the expression of osteoblast related genes and ALP levels. Fold change in the gene expression of **(A**) Runx-2 and (**B**) osteocalcin in cells cultured in filtered or non-filtered medium over a period of 12 days was investigated. On day 2, a significant fold change (*p < 0.05) in the expression of Runx-2 was observed when cells were cultured in Mg100 non-filtered medium compared to osteogenic medium. On day 4, a significant fold change (**p < 0.01) in osteocalcin expression was also observed when cells were cultured in Mg100 non-filtered medium compared to Mg100 filtered medium and osteogenic medium. On day 8 and 12, a similar trend was also observed for osteocalcin expression (*p < 0.05). (**C**) The expression of COL1A1 at day 2 was significantly downregulated when cells were cultured in non-filtered medium compared to filtered medium (*p < 0.05) and osteogenic medium (^#^p < 0.001). Fold change is relative to the control, represented by a fold change of 1. (**D**) The presence of ALP protein was evaluated at different time points (day 2, 7, 12) following culture in Mg 100 filtered and non-filtered medium. On day 12, a significant increase (^#^p < 0.001) in the presence of ALP was observed when cells were cultured in Mg100 non-filtered medium compared to Mg100 filtered medium and control. However, a significant reduction (^#^p < 0.001) in ALP presence was also observed when cells were cultured in Mg100 non-filtered medium compared to osteogenic medium. ALP protein was normalised to DNA concentration. Osteogenic medium was used as the positive control. The bars represent the mean and standard deviation in the positive orientation of three independent experiments, each with n = 3.

Gene and protein studies were performed to determine the effect of the corrosion products on osteogenic lineage differentiation. A significant increase (p < 0.05) in Runx-2 expression was observed when cells were cultured in osteogenic medium compared to cells cultured in Mg100 non-filtered medium (Fig. 6A). On the other hand, osteocalcin gene expression was significantly enhanced when cells were cultured in Mg100 non-filtered medium compared to cells cultured in osteogenic medium and Mg100 filtered medium on days 4, 8 and 12 (Fig. 6B). On day 4, a significant (p < 0.01) change (3x fold) in osteocalcin expression was observed when cells were cultured in Mg100 non-filtered medium compared to osteogenic medium and Mg100 filtered medium. The same trend was also observed on day 8 and 12 (p < 0.05). The effect of Mg100 non-filtered medium started to diminish by day 12. On day 2, COL1A1 expression was reduced when cells were cultured in Mg100 non-filtered medium compared to Mg100 filtered medium (p < 0.05) and osteogenic medium (p < 0.001). Levels of alkaline phosphatase (ALP) (a hydrolase enzyme with an integral role in providing free phosphates for the synthesis of calcium phosphate) were significantly higher (p < 0.001) in cells cultured in Mg100 non-filtered medium compared to Mg100 filtered medium and control on day 12. However expression was significantly lower (p < 0.001) compared to cells cultured in osteogenic medium (Fig. 6C).

### The effect of Mg conditioned media on RAW cell metabolic activity

**Figure 7:**
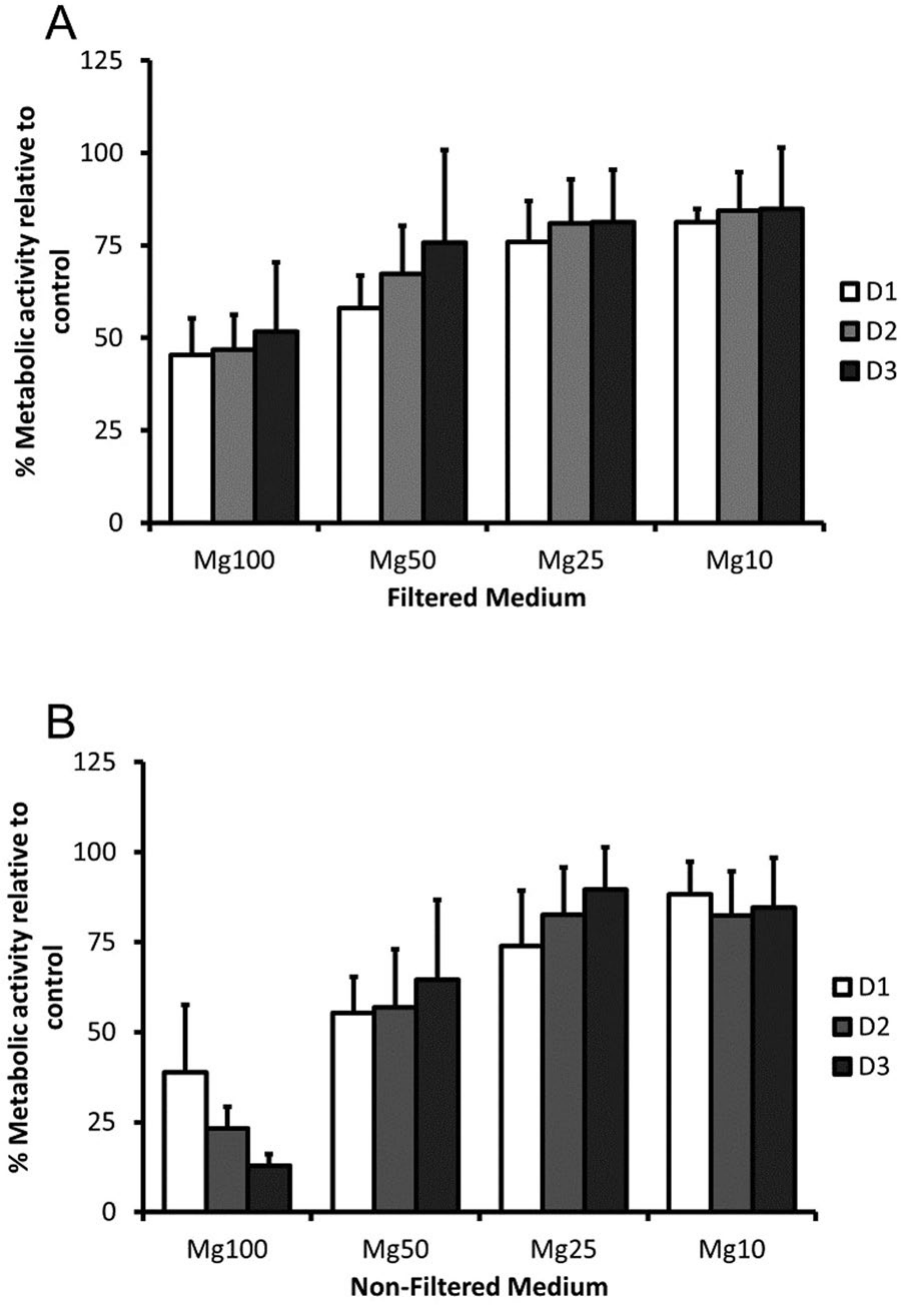
The effect of Mg conditioned medium on RAW cell metabolic activity was investigated using AlamarBlue assay. RAW cells were cultured in the presence of various concentrations of Mg conditioned medium, **(A**) filtered medium and **(B)** non-filtered medium. A reduction in metabolic activity was observed when cells were cultured in both Mg100 filtered and non-filtered medium over the 3 day culture period compared to the control, however culture in Mg100 non-filtered medium had the greatest effect. Culture in Mg50 filtered and non-filtered medium also resulted in a reduction in metabolic activity at day 1 and 2 compared to the control. The data is presented relative to the control (100%). The bars represent the mean and standard deviation in the positive orientation of three independent experiments, each with n = 3.

The response of pre-osteoclast cells (RAW cells) to the presence of Mg conditioned media was investigated. Figure 7 shows a dose-dependent effect of Mg conditioned media on RAW cell metabolic activity. The reduction of Mg conditioned media concentration (filtered and non-filtered media) resulted in increased metabolic activity. A reduction in metabolic activity was observed when cells were cultured both in Mg100 filtered and Mg100 non-filtered medium over the 3-day culture period compared to the control. With Mg50 filtered medium, a reduction in metabolic activity was observed at day 1 and 2 compared to the control and a similar trend was observed when cells were cultured in Mg50 non-filtered medium. Treatment duration and concentration of Mg conditioned media significantly affected the behaviour of RAW cells. Over time cells were able to adapt to the presence of Mg conditioned media and metabolic activity increased. However, in the presence of corrosion granules, time did not restore the metabolic activity of cells cultured in Mg100 non-filtered medium (Fig. 7B).

### Osteoclastogenesis related gene analysis on RAW cells after culture in Mg conditioned media

**Figure 8:**
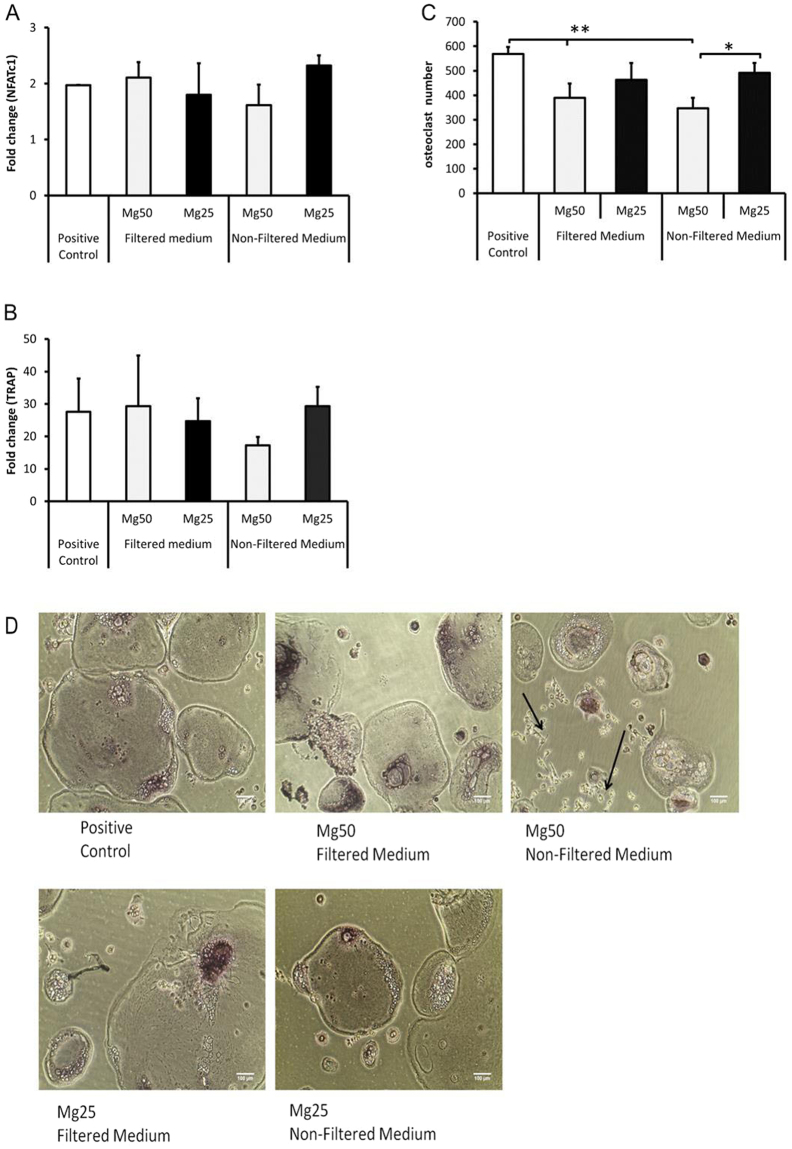
Effect of Mg conditioned media on osteoclast related genes. RAW cells were cultured in the presence of Mg conditioned medium at varying concentration with (non-filtered medium) or without (filtered medium) the presence of corrosion granules for a period of 5 days. Fold change in the expression of (**A)** TRAP and (**B)** NFATC-1 after treatment with Mg conditioned medium was investigated. No significant difference in gene expression was detected between the treated conditions and positive control. (**C)** TRAP assay was performed following culture in Mg conditioned medium in the presence of RANK-L. All TRAP positive multinucleated cells in each well were counted for analysis. Culture in Mg50 filtered and non-filtered medium resulted in a significant reduction in cell number (**p < 0.01) compared to the positive control. A significant difference (*p < 0.05) in cell number was also observed in cells cultured in Mg50 non-filtered medium compared to Mg25 non-filtered medium. The bars represent the mean and standard deviation in the positive orientation of three independent experiments, each with n = 8. The fold change is relative to the untreated sample (without RANK-L or Mg). Positive control represents RAW cells cultured in normal growth medium and then treated with RANK-L to induce differentiation. (**D)** Representative images of TRAP positive multinucleated cells taken after 5 days of culture are presented. The arrow represents undifferentiated RAW cells. Images from three independent experiments were analysed, each with n = 8.

The effect of Mg conditioned media on the expression of osteoclast specific markers (NFATC-1 and TRAP) was investigated. The culture of RAW cells with Mg filtered and non-filtered medium did not alter TRAP and NFATC-1 gene expression (Fig. 8A,B). However, the treatment with both Mg50 filtered and non-filtered medium significantly reduced (p < 0.01) total osteoclast-like cell numbers compared to the positive control (Fig. 8C). There was a significant increase (p < 0.05) in osteoclast-like cell formation when cells were cultured in Mg25 non-filtered medium compared to Mg50 non-filtered medium. The presence of corrosion products (Mg50 non-filtered medium) appeared to result in the formation of smaller osteoclast-like cells compared to the positive control (Fig. 8D).

### The response of mature osteoclast cells to Mg non-filtered conditioned media

**Figure 9:**
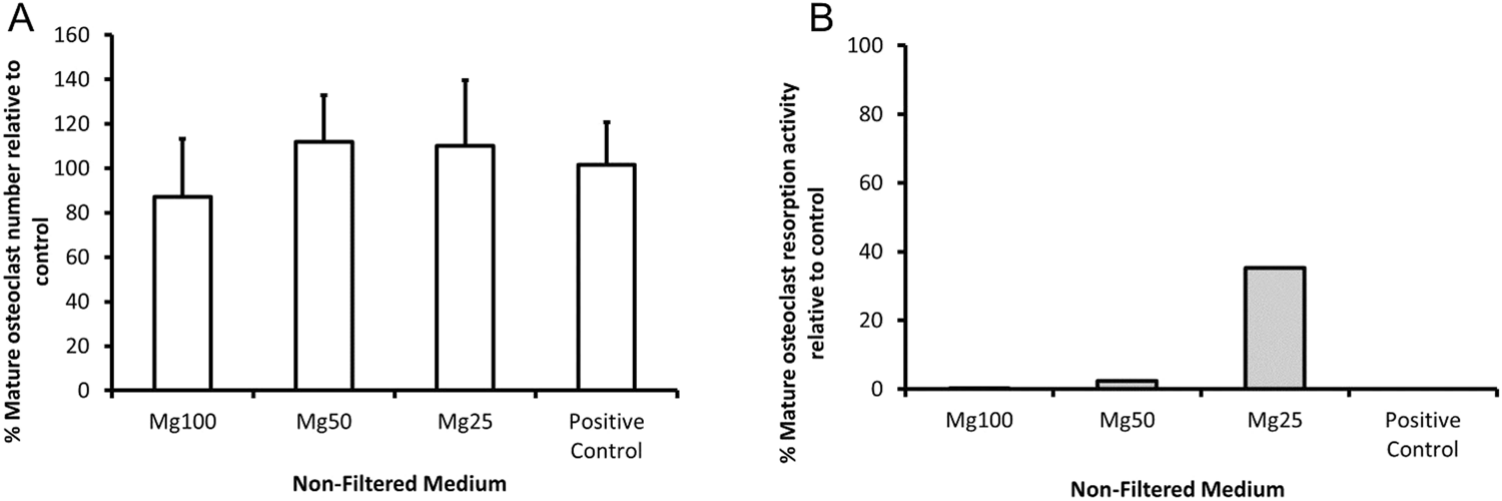
Effect of Mg non-filtered medium on mature osteoclast activity. (**A**) The number of TRAP positive mature osteoclast cells present on bone slices following a 24 hr treatment with various concentration of Mg non-filtered conditioned medium is presented. Mature osteoclast cell number was normalised relative to the standard control (100%). (**B**) Resorption pits formed by mature osteoclasts in the presence of Mg non-filtered medium were counted and normalised to osteoclast cell number. Resorption activity was normalised relative to the standard control (100%). Cells cultured in MO standard medium with the presence of sCT (inhibitor of resorption activity) were used as the positive control. The bars represent the mean and standard deviation in the positive orientation of three independent experiments, each with n = 6.

Further investigation on the effect of Mg conditioned media on mature osteoclast resorption activity was performed and results illustrated in Fig. 9. Treatment with various Mg non-filtered medium concentrations did not affect osteoclast cell number. The treatment with calcitonin (sCT), an inhibitor of osteoclast activity did not affect cell number either. Since the cells were able to tolerate the various concentrations of Mg conditioned media, the resorption activity of these cells was also investigated. The presence of Mg non-filtered conditioned medium resulted in a reduction in osteoclast resorption activity. Even at low concentration (Mg25), the activity was low compared to the control. The culture of cells in Mg100 non-filtered medium and the presence of calcitonin completely inhibited the activity of mature osteoclast cells.

## Discussion

In the draft of the new American Society for Testing and Materials (ASTM) standard (ASTM WK52640), it has been pointed out clearly, that the experimental approaches informing development of standards for application of Mg as biodegradable metal implants must control the corrosion test environment through standardization of conditions and utilization of physiologically relevant electrolyte fluids. One of the most important step in *in vitro* testing is the preparation of extracts from the implants, and current testing standards requires the removal of the granules formed during the corrosion process. Other studies^13,14^ have reported acceptable biocompatibility of Mg following *in vitro* testing using Mg extracts (containing only soluble corrosion products). However, to emulate the biological response to the corrosion products in the *in vivo* environment, the use of complete extracts from Mg metal conditioned medium containing both the soluble and insoluble (granule) corrosion products was preferred in this study. The corrosion of Mg resulted in the release of Mg^2+^ into the surrounding medium, the higher the corrosion rate the higher the amount released in a unit of time, but ultimately the medium would become saturated with the deposition of corrosion granules. During the corrosion of Mg metal, calcium phosphates were also formed through the consumption of calcium and phosphate ions from the surrounding medium as confirmed by the chemical analysis here and elsewhere by other authors^8,15,16^. The calcium phosphates formed were believed to be most likely amorphous calcium deficient hydroxyapatite as indicted by the low Ca/P ratio, hence it was undetectable by XRD as shown in our previous work^17^. The *in vitro* corrosion model presented here predicts the local cellular response to Mg corrosion in a manner analogous to the concentration gradient effects of corrosion products in the surrounding tissue *in vivo*. This study has demonstrated that the *in vitro* response of mesenchymal stem cells and cells in osteoclastogenic lineage depends on the concentration of the Mg corrosion products. In addition, the presence of corrosion granules has profound effects on cell proliferation and maturation of differentiated cells.

### Effects on cellular and subcellular activities of hMSCs

hMSCs derived from bone marrow were able to tolerate Mg ion concentration of up to 16 mM, which is 16 times greater than that typically found in serum (0.70–1.0 mM), but metabolic activity and proliferation was enhanced only at concentrations ≤10 mM. The mere presence of corrosion granules resulted in reduced metabolic activity and proliferation, and more so in the presence of high corrosion granule concentration (Mg100). The effect was also time dependent, with longer exposure to the corrosion granules resulting in reduced cell viability. When cells were presented with Mg100 non-filtered medium, no change in cell number was observed as shown by the DNA concentration analysis. Indeed when corrosion granules were reduced (Mg50 non-filtered medium) proliferation capacity was improved and further reduction in corrosion granule concentration resulted in enhanced cell proliferation, which was also the case for filtered medium. When cells were cultured in the presence of Mg non-filtered conditioned media, cell aggregates were formed. It is the first time that cellular response of hMSCs to the presence of Mg corrosion granules was captured in real time imaging and it was very intriguing that hMSCs co-ordinately migrated towards and cumulated those granules presented in 2D culture *in vitro*. Hence, further investigation on the fate of those granules was performed using TEM analysis to verify Mg presence at subcellular level. In the presence of corrosion granules, changes in the structure and distribution of organelles was observed. Examination of the treated cells showed a trend that mitochondria and organelles containing electron dense materials were distributed towards the surface of the cell. Considering the format of 2D cell culture, it would be reasonable to anticipate more transmembrane and subcellular activity for cell surface facing the corrosion products. This would explain the significant blebbing observed on part of the cell membrane where the corrosion granule was expected to be present. This could be explored further as blebbing is also associated with apoptosis.

Since the corrosion granules were composed of MgCO_3_^17^ and calcium phosphates^8^, it is believed the intracellular degradation of the corrosion granules also leads to the release of Ca^2+^, Mg^2+^ and phosphate ions. MgCO_3_ and calcium phosphates readily degrade inside the lysosomes due to the acidic environment^18^. Cells with a lot of clear space were also noticed and considering the rounded shape of those vacuoles it is very likely those were structures left behind following the degradation of endocytosed corrosion granules. The peripheral collection of highly vacuolated features has also been observed in hMSCs by Pasquinelli *et al*.^19^. Together with the presence of lysosomes as evidenced by the presence of electron dense bodies, the highly vacuolated features could be an indicator of intense endocytotic activity. In addition, the autolysosomes visible near altered cisternae and mitochondria further strengthen our hypothesis that treated cells were responding to the presence of large amounts of corrosion products. In order to maintain homeostasis, the autolysosomes were most likely responsible for the intracellular degradation and removal of old and damaged organelles due to the presence of high Mg corrosion products^20^. Organelles containing concentric lamellar features were also seen scattered at the periphery of the cells amongst the autolysosomes. These organelles were only seen in the treated sample; therefore it is believed that the presence of these autolysosomes was a consequence of the presence of Mg corrosion granules. It might be relevant to the endocytosis process or functions as a secretory organelle^21^.

The increase in expression of osteoblast related markers when cells were cultured in the presence of corrosion products could be related to a continuous supply of Mg ions afforded by the corrosion granules in the medium. Other studies^22–24^ have shown that the presence of Mg can induce osteogenic differentiation. It was observed in this study that the presence of corrosion granules was able to induce mRNA and protein expression of osteoblast markers such as osteocalcin and ALP. Osteocalcin is primarily produced by committed osteoblasts and signifies a mature osteogenic phenotype. This is not typically observed in differentiating MSCs until after at least 14 days of *in vitro* culture in the presence of osteogenic supplements^25^. In the current work, even though osteocalcin mRNA and ALP protein levels were upregulated when treated with Mg corrosion granules (Mg non-filtered medium), the expression of early upstream markers (Runx2 and COL1A1) were not significantly enhanced. Other studies have also reported inhibition of mineralisation in the presence of Mg^14,26^. Hence it is proposed that the presence of Mg corrosion granules induces the differentiation of hMSCs into a particular type of osteoblast cells, marked by the early expression of osteocalcin. Osteocalcin, usually used as a marker of bone activity, may also be involved in the recruitment and differentiation of osteoclast precursors, acting as an early signal corroborating molecular pathway of osteoclastogenesis^27,28^. Correlating with the evidence of TEM, collectively the data implies that the differentiated MSCs contribute directly to the clearance of corrosion particles via endocytosis.

### Effects on osteoclastogenesis

RAW cells were highly sensitive to the high concentrations of Mg conditioned media, particularly in presence of corrosion granules. In the presence of corrosion granules, metabolic activity was significantly reduced; similar to the observation with hMSCs. Even though metabolic activity was still significantly reduced in the presence of Mg conditioned media, RAW cells were able to adapt to the change in environment. The capability of pre-osteoclast cells to fuse into multinucleated osteoclast-like cells was reduced in the presence of Mg conditioned media, resulting in a large number of undifferentiated cells, despite the fact that the cells were treated with RANK-L. Also, there was no correlation between the number of multinucleated TRAP positive osteoclast-like cells and expression of osteoclast specific genes (TRAP, NFATc1). No significant differences in gene expression were observed between the different Mg concentrations and the positive control. This suggests that the low numbers in multinucleated osteoclast-like cells was due to the inhibition of fusion at the cell surface, hence Mg corrosion products did not affect pre-osteoclast differentiation at least at gene level as observed here. The extracellular environment plays an important role in the process of osteoclastogenesis; acidic environments have been reported to have the ability to induce or trigger fusion of macrophages^29^. Furthermore, acidic environments increase osteoclast activity and inhibit osteoblast activity and alkaline environment increase osteoblast activity and reduce osteoclast activity^5,30^. Zhai *et al*.^31^ also showed that both high Mg concentration and pH had an inhibitory effect on osteoclastogenesis. Therefore, the reduction in pre-osteoclast cellular activity could be due to the presence of high magnesium ions (>6 mM) or the alkaline environment (>pH8). The reduction of Mg ion concentration (<3 mM) and pH restored osteoclast cell formation to levels comparable to the control.

Interestingly mature osteoclasts were able to tolerate high concentrations of Mg conditioned medium compared to RAW cells. It was noticed that mature osteoclast cells were able to tolerate high Mg^2+^ concentration (Mg100) without affecting cell number. On the other hand, in the presence of Mg conditioned media the bone resorption activity of mature osteoclast cells was significantly reduced, which was also confirmed by calcitonin treated samples, an inhibitor of osteoclast activity. It’s been shown that osteoclast-mediated bone resorption activity is pH dependant, with pH 6.8 regarded as the optimum pH^32,33^. When the conditioned medium was diluted (thereby decreasing the pH), the activity of mature osteoclasts started to increase. The combination of low pH and availability of pro-resorptive factors (e.g. RANK-L) is essential for osteoclast mediated activity. Acidosis plays an important role in initiating osteoclast activity and upregulating factors associated with bone resorption (TNF, NFTAC and TRAP)^33^. Treatment of osteoclast cells with high magnesium chloride concentration has been shown to increase osteoclast cell activity^34^. The reduction in bone resorption activity observed here is likely due to the alkalinity of the conditioned medium rather than Mg ion concentration per se.

**Figure 10:**
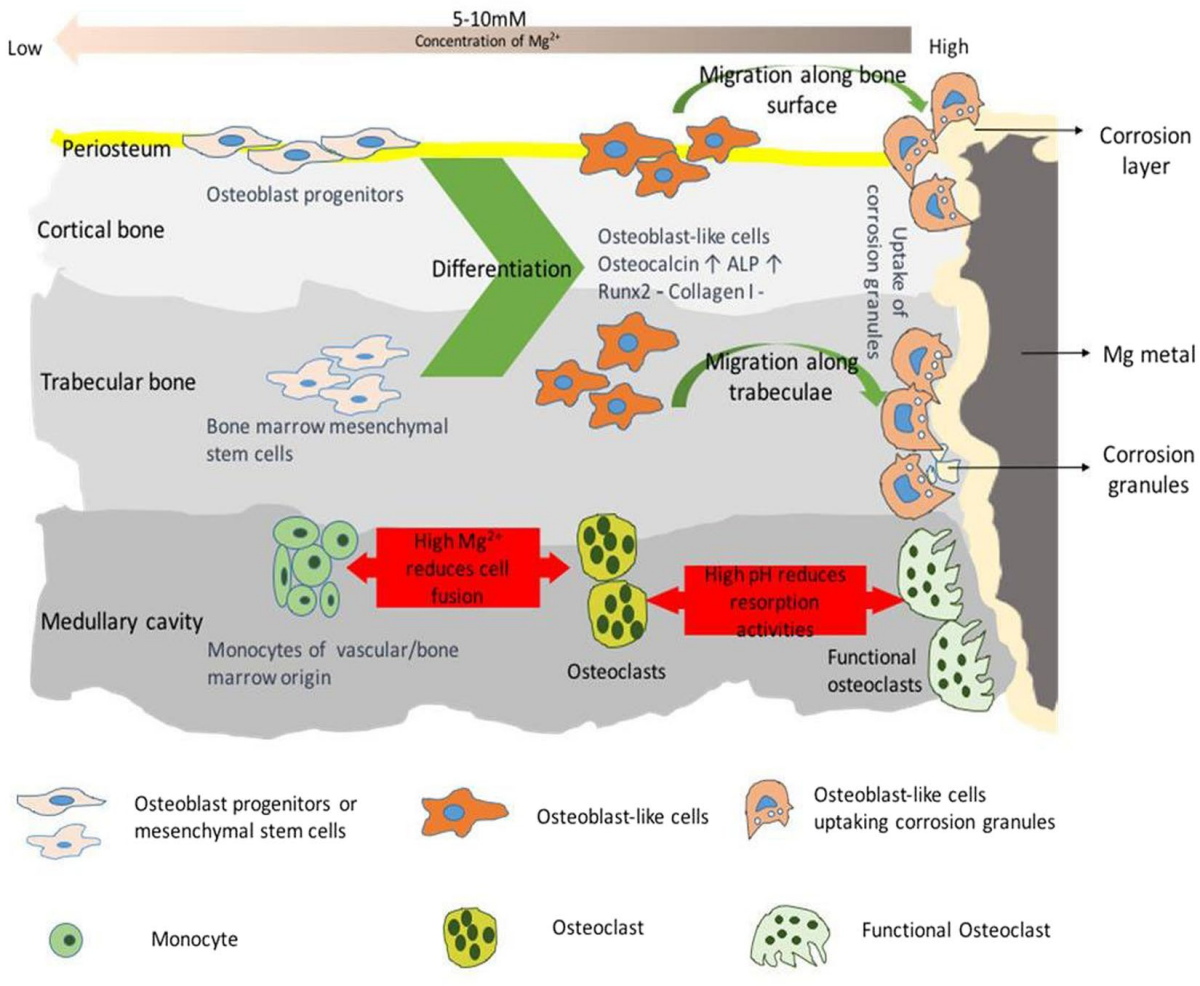
Summary of the cellular response. The effect of Mg corrosion products varies depending on the state of differentiation of cells and concentration gradients of Mg^2+^ in the surrounding tissue. High Mg corrosion granules indirectly inhibits bone healing by interfering with the differentiation process of MSCs in bone marrow and probably also osteo-progenitors in periosteum. MSCs and osteo-progenitors differentiate into osteoblast-like cells and migrate to the implantation site to remove the corrosion granules. On the other hand, the presence of high Mg ions reduces fusion of pre-osteoclast cells, thereby affecting osteoclastogenesis. Furthermore the presence of high pH caused by the fast degrading Mg metal reduces the resorption activity of mature osteoclast cells.

Based on our findings here, Fig. 10 illustrates our anticipation of the cellular response in the presence of a corroding Mg biomaterial *in vivo*. Where stem cells in bone marrow are in direct proximity to the corroding Mg metal, they experience a high concentration of the corrosion products. Stem cells respond by differentiating into osteoblast-like cells that act to clear the corrosion granules directly via endocytosis or indirectly by releasing osteocalcin which in turn recruits and activates osteoclasts to the implantation site. However with the function of the osteoclasts compromised, the activities of the differentiated MSCs are diverted from laying down new bone to clearing away the high concentration of the corrosion granules. Further away from the implantation site as the Mg ion concentration decreases to below 10 mM, the presence of the bioactive ions from the corrosion products induces the differentiation of MSCs to bone forming cells (osteoblasts) leading to enhanced bone formation. Furthermore the cell metabolic and proliferation activities for progenitors are enhanced, promoting bone formation and remodelling. This hypothesis could explain why Mg implant with high corrosion rate had shown histologically the presence of osteoblasts, but with no corresponding increase in bone volume around the implants^35^. The variation of corrosion behaviour with the location of the implant in bone observed *in vivo* may also suggest the contribution of osteoblasts and its progenitor cells to the removal of corrosion products^36,37^.

## Conclusions

Collectively, findings in this study indicated concentration-dependent effects of Mg corrosion products on cellular activity of bone marrow derived stem cells and on osteoclastogenesis *in vitro*. The effect of Mg corrosion products also varied depending on the state of differentiation of cells and length of exposure. The presence of the corrosion products significantly altered the cells’ metabolic and proliferative activities, which further affected cell fusion/differentiation. While cells tolerated higher than physiological range of Mg concentration (16 mM), concentrations below 10 mM were beneficial for cell growth. Furthermore, MSCs were shown to contribute to the clearance of intercellular corrosion granules, whilst high concentrations of corrosion products negatively impacted osteoclast progenitor cell number and mature osteoclast cell function.

## Electronic supplementary material

Mg50 non-filtered medium

Mg100 non-filtered medium
